# CD200R1 regulates eosinophilia during pulmonary fungal infection in mice

**DOI:** 10.1002/eji.201847861

**Published:** 2019-08-07

**Authors:** Samira Salek‐Ardakani, Thomas Bell, Christopher P Jagger, Robert J Snelgrove, Tracy Hussell

**Affiliations:** ^1^ National Heart and Lung Institute, Department of Inflammation Development & Repair, Imperial College London UK; ^2^ Manchester Collaborative Centre for Inflammation Research (MCCIR) Manchester UK

**Keywords:** CD200R1, *Cryptococcus neoformans*, eosinophilia, lung, Th2 immunity

## Abstract

CD200 receptor 1(CD200R1) signalling limits myeloid cell responses and reduces autoimmunity, alloimmunity and viral‐mediated immunopathology, but has never been examined in the context of eosinophilic inflammation. Susceptibility to lung fungal infection is associated with T‐helper 2 (Th2) cytokine dominated responses and strong eosinophilic pathology. Blockade of CD200R1 enhances type I cytokine responses in many infectious and non‐infectious settings and so may promote a more protective response to fungal infection. By contrast, we demonstrate that, rather than promoting type I cytokine responses, CD200R1 blockade enhanced eosinophilia in a mouse model of *Cryptococcus neoformans* infection, whereas CD200R1 agonism reduced lung eosinophilia – with neither strategy completely altering fungal burden. Thus, we reveal a surprising disconnect between pulmonary eosinophilia and cryptococcal burden and dissemination. This research has 2 important implications. Firstly, a lack of CD200R1 signalling enhances immune responses regardless of cytokine polarisation, and secondly reducing eosinophils does not allow protective immunity to develop in susceptible fungal system. Therefore, agonists of CD200R1 may be beneficial for eosinophilic pathologies.

## Introduction

The pleiotropic functions of myeloid cells require tight regulation to promote immunity to potentially harmful pathogens whilst limiting immunopathology. This regulation is achieved by both soluble factors and cell contact‐dependent interactions, for example those mediated through CD200 receptor (CD200R1). CD200R1 is type 1 transmembrane protein and member of the immunoglobulin superfamily that is predominantly expressed by cells of the myeloid lineage. Ligation with the more broadly distributed ligand, CD200, delivers a unidirectional inhibitory signal to the myeloid cell 1. Mice lacking CD200 display elevated numbers of activated macrophages and develop more aggressive autoimmune diseases 2, 3, suggesting that CD200 restrains the tonic activation of macrophages to promote homeostasis. Immunotherapeutic administration of CD200 alleviates excessive inflammation seen in auto‐ and alloimmune conditions 4, 5, 6.

Alveolar macrophages are the sentinels of the airways and determine the magnitude and orientation of the immune response 7. We have previously reported that these cells express high basal levels of CD200R1 that interacts with CD200 expressed on the luminal aspect of the airway epithelium 7. Alveolar macrophages are de‐regulated in naïve mice lacking CD200 8. Furthermore, during influenza viral infection, mice lacking CD200 clear virus more efficiently, but develop pronounced immunopathology that is slower to resolve. Restoration of CD200 signalling returns inflammatory cell control. The CD200‐CD200R1 axis therefore balances pathogen clearance and inflammation 9, 10.

CD200R1 has predominantly been studied in the context of Th1‐driven inflammatory diseases/infections 9, 11, 12, 13. In fungal infection, Th2 responses are generally deleterious, but the influence of CD200R1 is not known. *Cryptococcus neoformans* is an encapsulated budding yeast causing disease in immunocompetent individuals, but immunologically compromised are at greatest risk 14, 15, 16. Failure to control fungal burden results in the lung results in dissemination to extra‐pulmonary tissues, including the brain (cryptococcosis). In recent years clinical cases of cryptococcosis have escalated and in the AIDS infected population 6–8% develop cryptococcus‐associated meningitis 17, 18. Whilst a strong Th1 response is critical in resistance to *C. neoformans*, a Th2‐driven eosinophilia is observed in the bronchoalveolar lavage (BAL) fluid of some patients, and hypereosinophilia reported with disseminated cryptococcal disease 19. Similarly, in mouse models of *C. neoformans* infection, resistant mice (CBA, C.B‐17, BALB/c) generally produce higher concentrations of type 1 cytokines 20, 21, 22. In contrast, susceptible strains (C57BL/6, C3H and B10.D2) develop a Th2 driven pulmonary eosinophilia where up to 40% of airway cells are eosinophils 23, 24. This response is non‐protective and results in tissue damage resulting from degranulation and crystal deposition by eosinophils 20.

The role of CD200R1 signalling in fungal infection and, more broadly, Th2‐focused inflammation is yet to be determined. As blockade of CD200R1 enhances type I cytokine responses, we postulated that the response to fungal disease would switch from non‐protective to protective. Surprisingly, a loss of CD200R1 actually enhanced Th2 responses and pulmonary eosinophilia, but had no effect on fungal burden or dissemination. Conversely, agonists to CD200R1 reduced pulmonary eosinophilia without impacting on fungal clearance. CD200R1 therefore alters the magnitude, but not phenotype of fungal lung disease. These studies also highlight a clear disconnect between *C. neoformans* clearance and pulmonary eosinophilia. Thus, protection from fungal lung disease may not occur by eliminating Th2‐associated eosinophils, but rather specific induction of a Th1 program of inflammation.

## Results

### Alveolar macrophages are the predominant CD200R1 expressing cells during *C. neoformans* infection

**Figure 1:**
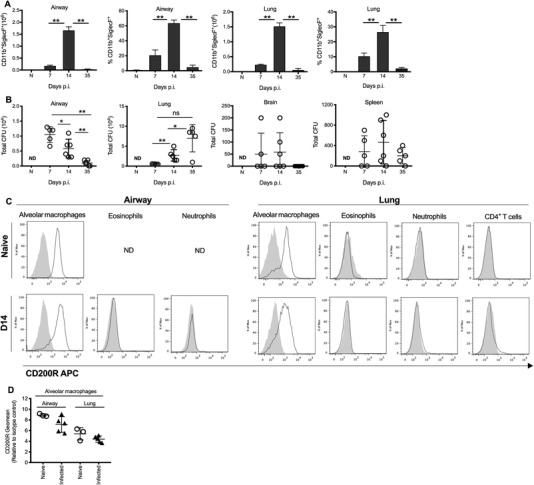
Alveolar macrophages are the predominant cell type expressing CD200R during *C. neoformans* infection. Wild‐type mice were infected intranasally with *C. neoformans* (strain 52D). Total numbers and proportions of eosinophils in the airways and lung (A) were determined by flow cytometry. Total fungal CFU in the airways, lung tissue, brain and spleen (B) were determined by plating out on selective agar. CD200R1 expression on cells isolated from airways and the lung tissue (C and D) of wild‐type mice were analysed by flow cytomtery at day 0 and 14 post infection with *C. neoformans*. Histogram overlay depicting the levels of CD200R1 expression on alveolar CD11c^+^ macrophages, eosinophils (CD11c^−^CD11b^+^SiglecF^+^), neutrophils (CD11c^−^CD11b^+^Ly6G^+^) and CD4^+^ T cells. ND, not detected, gray: isotype control, black: CD200R1 expression. (**D**) CD200R expression levels (by relative geometric mean of the stain and its respective isotype control) on alveolar CD11c^+^ macrophages were measured in the airways and the lung tissue of naïve mice (*n* = 3) and mice infected with *C. neoformans* (*n* = 5). Open circle, naive; Closed triangle, infected. Data shown represent 3–5 mice per group from one experiment and are representative of three independent experiments. Data shown as mean ±SEM. Statistical significance was determined by Mann–Whitney *U*‐test, *p < 0.05; **p < 0.01, ***p < 0.001.

The *C. neoformans* susceptible C57BL/6 mouse model was utilized to dissect the role of CD200R1 in modulating Th2 inflammation and fungal clearance. *C. neoformans* infection induced cellular infiltration into the airways and lung parenchyma, which peaked at 14 days, but persisted 35 days after infection. Eosinophils were the predominant cell type in the airways and lung tissue (Fig. 1A). As previously described, this Th2‐driven inflammation does not fully resolve fungal infection, with persistence in the airways and lung at 35 days post infection and dissemination to the brain (Fig. 1B).

As previously reported 8, a high level of CD200R1 expression was detected on alveolar CD11c^+^ macrophages in the airways and lungs of naïve mice, with expression remaining high upon *C. neoformans* infection (Fig. 1C and 1D). Eosinophils, neutrophils and CD4^+^ T cells were devoid of CD200R1 at homeostasis and upon *C. neoformans* infection (Fig. 1C), suggesting that any impact of CD200R1 manipulation in the context of fungal infection was likely mediated through macrophages (refer to Supplementary Fig. 1 for flow cytometry gating strategy).

### CD200R1 limits eosinophilic inflammation

**Figure 2:**
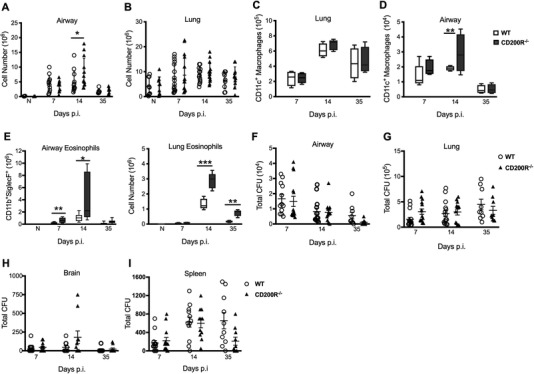
A Lack of CD200R1 exacerbates eosinophilia inflammation during fungal infection without altering pathogen clearance. Wild‐type and CD200R1^−/−^ mice were infected intranasally with *C. neoformans*. The total cell numbers in the airways (A) and lung tissue (B) were measured at day 7, 14 and 35 post infection. Total alveolar CD11c^+^ macrophages in the lung (C) and airways (D) were analysed by flow cytometry. Total eosinophils in the airways and in the lung (E) were analysed by flow cytometry. Data presented as box and whisker plots with median. Open box, wild‐type; filled box, CD200R1^−/−^ (C‐E). Total CFUs were determined by plating out BAL fluid (F), lung (G), brain (H) and spleen (I) homogenates from wild‐type and CD200R1^−/−^ mice. Open circle, wild‐type; Closed triangle, CD200R1^−/−^ (A, B, F‐I). Figures present data from 3 independent experiments with 4–5 mice per group in each experiment. Statistical significance was determined by Mann–Whitney *U*‐test, *p<0.05; **p<0.01, ***p<0.001.

We anticipated that removal of CD200R1 would enhance immunity and *C. neoformans* clearance. As expected, the cellularity of the airspaces increased in *C. neoformans* infected mice. An absence of CD200R only caused a mild increase in cellularity at day 14 after infection. (Fig. 2A). Total cell numbers and total numbers of alveolar macropahges in the lung parenchyma were comparable between CD200R1^−/−^ and littermate controls (Fig. 2B and 2C). Despite the similarity in total lung cellularity, total numbers of alveolar CD11c^+^ macrophages in the airways were raised in CD200R1^−/−^ mice during *C. neoformans* infection (Fig. 2D), as we have previously reported on Th‐1 and Th‐17 skewed pulmonary infection models^11^. Together, this data re‐enforces our assertion that the primary alteration may lie in the CD200R^hi^ macrophage compartment. The dominant effect in CD200R1^−/−^ mice during *C. neoformans* infection however, was the striking increase of airway and lung parenchyma eosinophils (Fig. 2E). This could not be explained by an increase in fungal burden since CFUs in the airways (Fig. 2F) and lung tissue (Fig. 2G) were comparable between wild‐type and CD200R1^−/−^ mice. Fungal dissemination to the brain and spleen (Fig. 2H and 2I) was also unaffected. Thus an absence of CD200R1 signalling seemingly augmented eosinophilc pathology in C57BL/6 mice, without a significant improvement in fungal clearance.

### Elevated type 2 cytokines in *C. neoformans* infected CD200R^−/−^ mice

**Figure 3:**
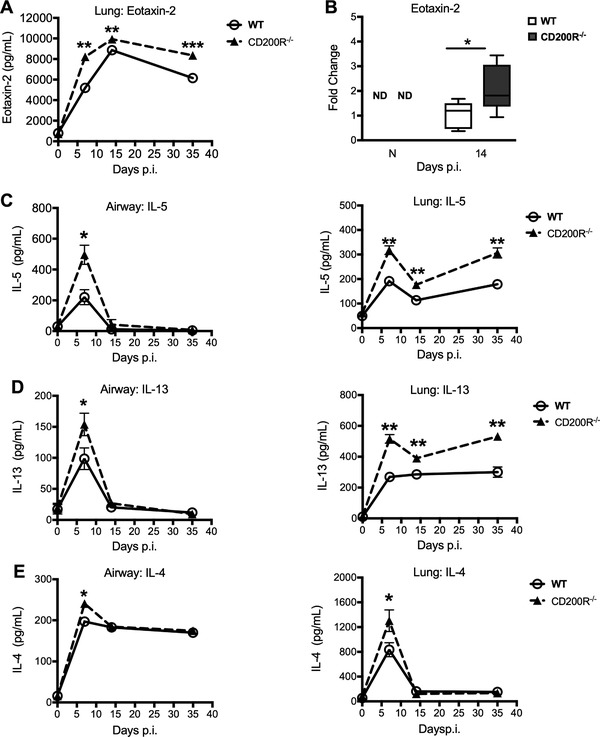
CD200R1^−/−^ mice exhibit augmented type 2 cytokines following *C. neoformans* infection. Wild‐type and CD200R1^−/−^ mice were infected intranasally with *C. neoformans*, and levels of eotaxin‐2 (A), IL‐5 (C), IL‐13 (D), IL‐4 (E) in the airways and lung homogenate were assessed by ELISA at indicated times post infection. Alveolar macrophages were isolated from the lung following 14 days post infection by fluorescence‐activated cell sorting and the fold changes in eotaxin‐2 mRNA levels (B) were assessed by Q‐PCR. Data show results for five mice per group from one experiment and are representative of two independent experiments. Solid Line, wild‐type; dashed line, CD200R1^−/−^ mice. Data shown as mean ±SD and statistical significance was determined by student's unpaired *t*‐test. *p<0.05; **p<0.01, ***p<0.001.

To determine why eosinophils were raised in CD200R1 knockout mice during *C. neoformans* infection, eotaxin‐2, a potent eosinophil chemoattractant, primarily derived from alveolar macrophages, was measured. Eotaxin‐2 protein (Fig. 3A) and mRNA in sorted alveolar macrophages (Fig. 3B), was elevated in *C. neoformans* infected CD200R1^−/−^ mice. Neutralization of eotaxin‐2 in wild‐type mice reduced airway (Supplementary Fig. 2A and B) and lung (Supplementary Fig. 2C and D) eosinophils, highlighting the importance of this chemokine in driving eosinophilia in this model. Similarly, eotaxin‐2 neutralization had no impact on fungal burden in the airways or the lung of treated mice (Supplementary Fig. 2E and F), reinforcing the concept that there is a disconnect between eosinophilia and fungal control. Similarly, the eosinophil survival factor IL‐5 (Fig. 3C), and other classical type 2 cytokines IL‐13 (Fig. 3D) and IL‐4 (Fig. 3E) were raised in the airways and lung homogenate of CD200R1*^−/−^* animals.

**Figure 4:**
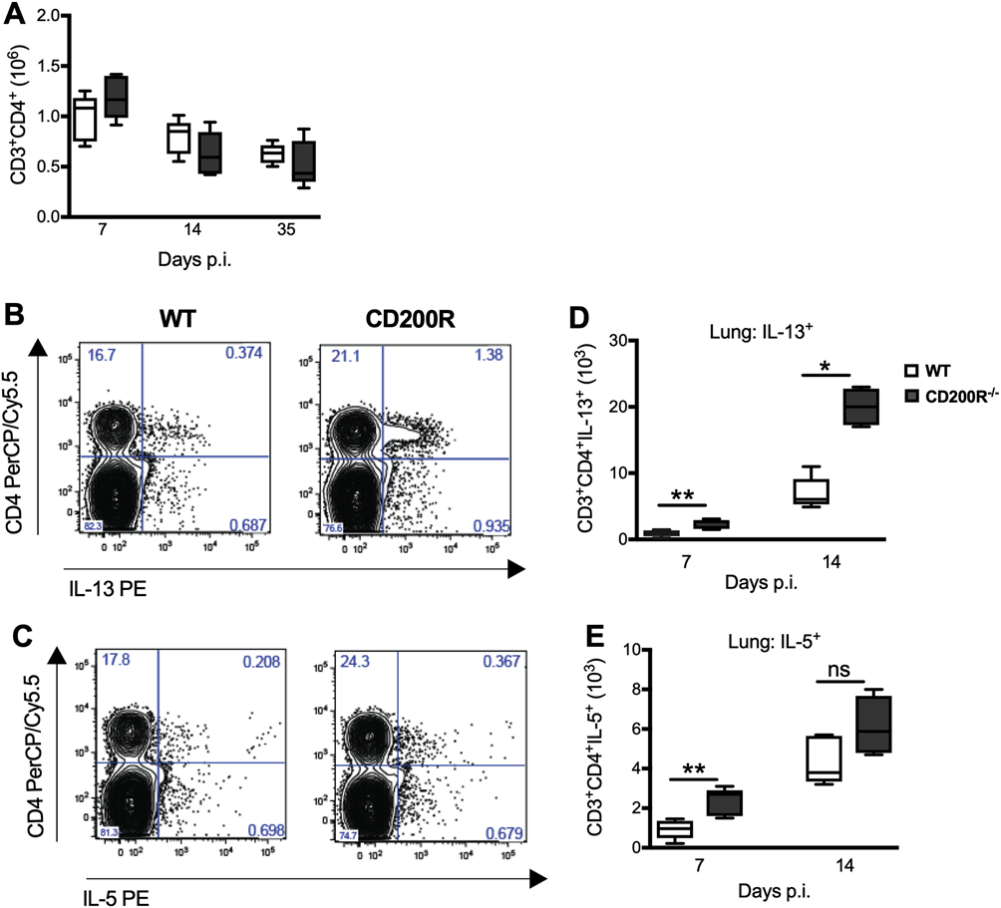
Heightened Th2 cytokine production by CD4^+^ T cells derived from CD200R1^−/−^ mice post fungal infection. Lungs were isolated from *C. neoformans* infected wild‐type and CD200R1^−/−^ mice at day 7 and 14 post infection. (A) Total number of CD4^+^ T cells were determined by flow cytometry. Representative plots are shown of lung CD4^+^ T cells cells versus IL‐13 (B) or IL‐5 (C) expression in wild‐type compared with CD200R1^−/−^ mice at day 14 post infection. The total number of CD4 lymphocytes expressing IL‐13 (D) or IL‐5 (E) in the lung was calculated by multiplying % of positive cells (as determined by flow cytometry) by total number of viable cells. Open box, wild‐type; filled box, CD200R1^−/−^. Data presented as box and whisker plots with median. Data shown represents five mice per group from one experiment and is representative of two independent experiments. Statistical significance was determined by Mann–Whitney *U*‐test,. *p<0.05; **p<0.01.

Whilst macrophages are the likely source of elevated eotaxin‐2 26, 27, the other elevated cytokines may derive from another cellular source 28, 29, 30. We observed no differences in total numbers of CD4^+^ T cells between *C. neoformans* infected CD200R1^−/−^ and wild‐type mice (Fig. 4A). However, intracellular cytokine staining revealed that the CD4^+^ T cells present produced more IL‐13 (Fig. 4B and 4D) and IL‐5 (Fig. 4C and 4E) in CD200R1^−/−^ mice.

### *C. neoformans* infected CD200^−/−^ mice also exhibit enhanced eosinophilic inflammation

C.

**Figure 5:**
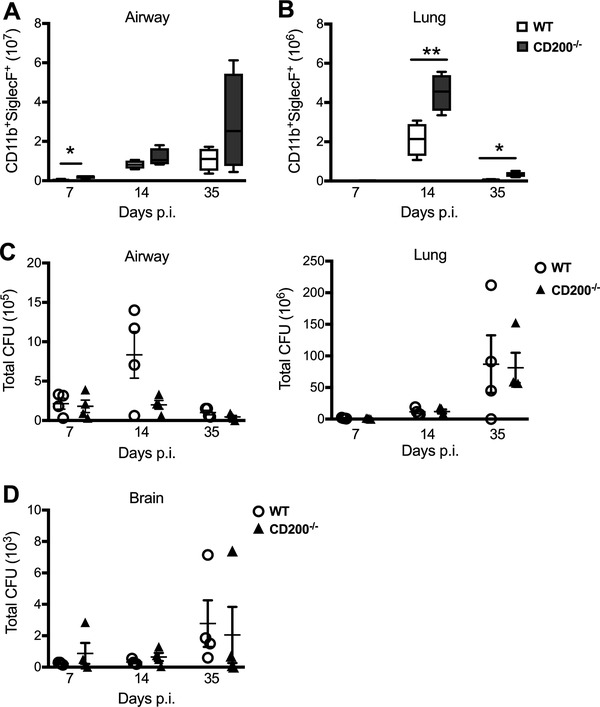
*C. neoformans* infected CD200^−/−^ mice also exhibit an augmented eosinophilic inflammation. Wild‐type and CD200^−/−^ mice were infected i.n with *C. neoformans*. Total numbers of eosinophils in the airways (A) and lung tissue (B) of wild‐type and CD200^−/−^ infected mice were determined by flow cytometry. Open box, wild‐type; filled box, CD200^−/−^. Data presented as box and whisker plots with median. Total CFUs were determined by plating out BALF and lung (C) and brain (D) from wild‐type and CD200^−/−^ mice. Open circle, wild‐type; closed triangle, CD200^−/−^. Data shown represents 5 mice per group from one experiment and is representative of two independent experiments. Statistical significance was determined by Mann–Whitney *U*‐test, *p<0.05; **p<0.01.

The interaction of CD200R1 with CD200 produces a unidirectional inhibitory signal to the CD200R1 bearing cell. We therefore reasoned that an absence of CD200 should result in a similar outcome as CD200R1^−/−^ mice during lung fungal infection. However, it is now acknowledged that there are four murine CD200R isoforms (R1‐R4), the precise functions of which are still poorly defined. Furthermore, uncertainty persists as to whether CD200 is the natural ligand for all receptors 3, 21, 22. To validate our observations seen in CD200R1^−/−^ mice and identify any potential ligand redundancy, mice deficient in CD200 were also analysed for their response to *C. neoformans*. Indeed, mice lacking CD200 displayed greater airway (Fig. 5A) and lung (Fig. 5B) eosinophils relative to wild‐type controls, although the phenotype was far more dominant in the lung tissue. Once again, there was no significant reduction in fungal clearance in the airways, lung tissue (Fig. 5C) or brain (Fig. 5D) of CD200^−/−^ mice, although the CFUs were trending down within the airways.

### Targeting the CD200‐CD200R axis during *C. neoformans* infection modulates eosinophilic inflammation

**Figure 6:**
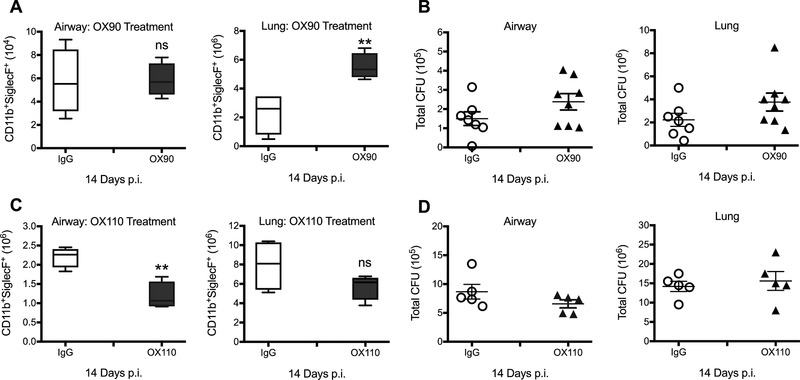
Therapeutic manipulation of the CD200‐CD200R1 axis during *C. neoformans* infection demonstrates the potential to harness eosinophilc inflammation. Wild‐type mice were treated with CD200 antagonist OX90 or IgG control antibody a day prior to infection and every 2 days post infection (A and B). Total eosinophils in the airways and lung tissue (A) were measured by flow cytometry at day 14 p.i. Fungal CFUs were enumerated in the BALF and lung homogenate (B) by plating on selective agar. Other groups of mice were treated with CD200R1 agonist, OX110, or IgG control (C and D). Total eosinophils in the airways and lung tissue (C) were analysed by flow cytometry at day 14 post infection. Fungal CFUs were enumerated in the BALF and lung homogenate (D) by plating on selective agar. Open circle, IgG treated mice; closed triangle OX90 or OX110 treated mice. Data shown as box and whisker plots with median. Data shown represents 5–8 mice per group from one experiment and is representative of two independent experiments. Statistical significance was determined by Mann–Whitney *U*‐test, *p<0.05; **p<0.01; ns, not significant.

To exclude additional, inherited defects in CD200/R1^−/−^ and CD200^−/−^ animals at steady state, we administered a CD200 antagonistic antibody (OX90) to *C. neoformans* infected wild‐type mice and once again observed increased lung eosinophils in treated animals (Fig. 6A). Furthermore and in keeping with our previous findings, no differences were observed in fungal clearance from the airways and lung tissue (Fig. 6B).

If an antagonist increases eosinophils, then an agonistic antibody to CD200R1 (OX110) should ameliorate inflammation and associated pathology. OX110 administration to *C. neoformans* infected mice reduced eosinophilic inflammation in the airways, but not the lungs (Fig. 6C) at day 14 post infection. Again, manipulation of the CD200R1 pathway had no significant impact on fungal clearance from the airways or lung tissue (Fig. 6D).

## Discussion

The discovery of CD4^+^ T cell subsets with polarised expression of specific cytokines has transformed our understanding of what constitutes a protective response and explains the diverse outcome on infection with the same pathogen in mice of different haplotype. Th‐2 type cytokine responses to the majority of viral, bacterial and fungal pathogens constitute a non‐protective response. Most in vivo models of inflammation show that BALB/c (H‐2^d^) mice respond with a more Th2‐cytokine response whereas C57BL/5 (H‐2^b^) mice are more prone to Th1‐cytokines. The murine models of *C. neoformans* infection, however, causes “non‐protective”, Th2‐driven eosinophilic lung pathology (the extent of which depends on T1/ST2 expression 23) and fungal meningitis in C57BL/6 mice, whereas BALB/c mice are non‐eosinophilic and relatively protected. By manipulating CD200R1 activity, we show that it critically regulates the amplitude of eosinophilia and type 2 cytokines, but has no significant impact on cryptococcal burden or dissemination. Eosinophils have previously been reported to phagocytose opsonised *C. neoformans* and wall off the fungus in peri‐vascular eosinophilic cuffs lining the alveolar space 24. Despite enhanced eosinophilia in CD200R^−/−^ mice, fungal burden was not significantly altered, which is in contrast to other studies where a reduction in eosinophilia (for example in surfactant protein D, Scavenger Receptor A, NADPH oxidase or eosinophil knockout mice) 25, 26 is associated with reduced fungal burden.

This outcome is evident in human disease where susceptibility to HIV‐associated cryptococcal meningitis is not associated with the induction of an incorrect inflammatory response. For example, stimulation of peripheral blood CD4+ T cells from such patients with a fungal protein does not induce Th2‐associated cytokines, nor are they abundant in soluble form in the cerebrospinal fluid 27, 28, 29. Rather, susceptibility stems from a lack of type‐1‐associated cytokines (IFN, TNF, IL‐6 and MCP‐1), an observation supported by cryptococcal susceptibility studies in murine models 30, 31, 32. Not only are type 1 cytokine responses crucial for cryptococcal clearance, but they need to be present early. Decreased cryptococcal‐specific IFN‐γ responses 27, 33, 34 are associated with the condition HIV‐associated cryptococcal immune reconstitution inflammatory syndrome (CM‐IRIS) (for a review see 35, 36). The ingredients for this condition include HIV‐mediated suppression of immunity, cryptococcal infection (either sub‐clinical or clinical) and rapid recovery of immunity after commencement of anti‐retroviral therapy leading to inflammatory disease. However, if cryptococcal‐specific IFN‐γ is sufficient at the outset then the fungal load is more likely to be low enough to prevent inflammation upon immune restitution. The results presented here, and the clinical observations in HIV patients with cryptococcal meningitis imply that induction of type 1 cytokines irrespective of type 2 cytokine neutralisation may prove more beneficial. Modulating immune responses to a pro‐inflammatory profile has continually proven protective in the case of cryptococcal lung disease. For example, ligation of the late T cell co‐stimulator OX40 (CD134) 37, blockade of IL‐10 receptor 38 administration of type 1 IFNs 39 TLR9 agonism 40 infection with interferon‐gamma producing *C. neoformans* 41 enhance Th1‐ and/or Th17‐associated cytokines and improve *C. neoformans* clearance.

Though the amplitude of eosinophilia does not significantly impact on *C. neoformans* burden in our studies, the mechanism of increased amplitude may be relevant to other non‐infectious allergic lung diseases including asthma. The expression of CD200R1 was not detected on the surface of eosinophils implying that the increase in the numbers of this cell type is not a direct consequence of the removal of the inhibitory signal imparted by CD200. Instead, the raised eosinophilia we observed in the absence of CD200R1 likely arises from the enhanced levels of eosinophilic chemoattractant cytokines IL‐5 and eotaxin‐2. We therefore propose that enhanced eosinophilia is due primarily to increased eotaxin‐2 production from CD200R1^+^ airway macrophages and show by its neutralisation that eotaxin‐2 plays a non‐redundant role in eosinophilic inflammation in this model. Whilst macrophages are the likely source of the elevated eotaxin‐2, they are unlikely to represent a prominent source of IL‐13, IL‐5 and IL‐4 during *C. neoformans* infection, with previous studies highlighting the critical role of ILC2 and Th2 cells as source of these cytokines 42, 43, 44. Accordingly, we observed an increase in Th2 cytokine producing CD4^+^ T cells in *C. neoformans* infected CD200R1 knockout mice. It is feasible that the altered macrophage compartment in these knockout animals further promotes a Th2 response by releasing CCL17 and CCL22 chemokines known to recruit Th2 cells to the site of infection. Furthermore, we have previously demonstrated that an augmented T cell response to influenza infection was observed in CD200^−/−^ animals secondary to augmented macrophage / DC responses, potentially attributable to enhanced antigen presentation 8. This may also be true in the context of cryptooccal infection, but in an environment that would promote a Th2 skewed T cell response.

In conclusion, our study has identified a novel role for CD200R1 in eosinophil regulation and proposes that this potent inhibitory receptor can be targeted in vivo to regulate eosinophil‐dependent pathologies.

## Materials and methods

### Mice and pathogens

All animal procedures and care conformed strictly to the United Kingdom Home Office Guidelines under the Animals (Scientific Procedures) Act 1986 and the protocols were approved by the Home Office of Great Britain. Eight‐ to 12‐wk‐old female C57BL/6 (Harlan Olac, Bicester, UK), CD200R^−/−^ and CD200^−/−^ were kept in pathogen‐free conditions at Bio Safety level 2. *C. neoformans* strain 52D was obtained from the American Type Culture Collection (ATCC 24067) and maintained on Sabouraud dextrose agar (Beckton Dickson). For infection, a single colony of *C. neoformans* was resuspended in Sabouraud dextrose broth (1% neopeptone and 2% dextrose; Becton Dickson) and grown to stationary phase (48–72 h) in a rotating culture at room temperature. The cultures were washed in phosphate‐buffered saline (PBS), counted on a hemocytometer, and adjusted in sterile PBS to the desired infective concentration.

### Mouse infection and treatment

Mice were anesthetized using isoflurane and infected intranasally (i.n.) with 2 × 10^4^ CFU (Colony‐forming units) of *C. neoformans* in 50 µL of sterile PBS. In some experiments wild‐type C57BL/6 mice were injected intraperitneally (i.p) with 100 µg OX110 (rat IgG1 agonistic anti‐mouse CD200R), 100 µg of OX90 (rat IgG1 antagonistic anti‐mouse CD200), 500 µg Eotaxin‐2 monoclonal antibody (Rat igG2a Clone 106521, Research and Diagnostic Systems) or 100 µg control rat IgG (Serotec, UK) 1 day before and every other day following infection. Mice were sacrificed on indicated days by injection of 3 mg of pentobarbitone.

### Enumeration of *Cryptococcus neoformans*

Lungs and brain were homogenized by passage through 100‐µm cell strainers (BD Labware). A total of 100 µL of cell suspension from lung homogenate, brain homogenate, and BAL fluid were diluted in PBS and incubated at room temperature for 48 h on Sabouraud dextrose agar plates. The total CFU per sample was then determined (number of colonies x dilution factor x original cell suspension volume).

### Cell recovery and isolation

Bronchoalveolar lavage (BAL) cells were collected by inflating the lung 3 times with 1.5 ml of PBS via an intratracheal cannula and centrifuged for 5 min at 240 x *g*. The supernatant was stored at ‐80°C for cytokine quantification and the cell pellets were resuspended in 500 µl R10F (RPMI‐1640 supplemented with 10% foetal calf serum and 1% penicillin/streptomycin) for flow cytometry. Lung lobes were removed, shredded using scissors and digested at 37°C with agitation in the presence of 0.13 mg/ml Liberase III enzyme (Roche) and 50 µg DNAse I (Roche) for 30 min and the reaction stopped with 2mM EDTA. Digested lung tissue was disrupted into single cell suspensions by passage through a 100‐µM cell strainers and cell suspensions were centrifuged for 5 min at 240 x g. Red blood cells were lysed by resuspension of the pellets in ACK buffer (0.15 M ammonium chloride, 1 M potassium hydrogen carbonate and 0.01 mM EDTA, pH 7.2) for 3 min at room temperature. Cells were spun, washed and resuspended with R10F media. Cell viability was assessed by haemocytometer and trypan blue exclusion.

### Flow cytometry

Cells were stained for surface markers and analyzed by flow cytometry (Supplementary Fig. 1). All antibodies were purchased from BD Pharmingen (Oxford, UK), R&D systems (Abingdon, UK) or eBioscience, UK. In brief, for lymphocyte identification 1 × 10^6^ cells were stained using αCD3‐FITC (Clone 17A2), αCD4‐PerCP‐Cy5.5 (Clone RM4‐5). Alveolar macrophages (CD11c^+^ cells) in the lung and airways were identified using αCD11b‐PerCP‐Cy5.5 (Clone M1/70) and αCD11c‐APC‐Cy7 (Clone N418). For eosinophil (CD11b^+^CD11c^−^SiglecF^+^) and neutrophil identification (CD11b^+^CD11c^−^Ly6G^+^) αSiglecF‐PE (Clone E50‐2440) and αLy6G‐FITC (Clone 1A8) was used respectively. Expression of CD200R1 on different cell populations were identified using αCD200R‐APC (Clone OX110). All antibodies were diluted in phosphate‐buffered saline (PBS) containing 1% bovine serum albumin (BSA)/0.05% sodium azide (PBA). Cells were stained for 30 min on ice, washed with PBA, and spun for 5 min at 240 *x* g. After washing, cells were then fixed for 20 min at room temperature with 2% formaldehyde/PBS. Cells were then washed in PBA and data were acquired on BD FACS Canto II and analysed with the FlowJo (TreeStar, Ashland, OR) analysis programme.

To detect intracellular cytokines, 10^6^ cells/ml were incubated with 50 ng/ml PMA, 500 ng/ml ionomycin (Calbiochem, Nottingham, UK), and 10 µg/ml brefeldin A for 4 h at 37°C. Cells were then stained with αCD3‐APC, αCD4‐PerCP‐Cy5.5 with either IL‐5/IL‐13‐PE and fixed as before. After permeabilization with PBS containing 1% saponin/1% BSA/0.05% azide (saponin buffer) for 10 min, cells were incubated for 30 min with anti‐IL‐5‐PE or anti‐IL‐13 PE diluted 1/50 in saponin buffer. Cells were washed once in saponin buffer and once in PBA and data were acquired on BD FACS Canto II.

### Cytokine quantification

Concentrations of cytokines Eotaxin‐2, and IL‐5, IL‐13 and IL‐4 (eBioscience) in bronchoalveolar lavage (BAL) fluid and lung homogenates were measured using Duoset ELISA kits according to the manufacturer's instructions.

### Real‐time quantitative PCR

Purified alveolar macrophages were lysed using RLT buffer (Qiagen, UK) and RNA extracted by Qiagen RNeasy mini kit (Qiagen, UK) as per manufacturer's instructions. cDNA was generated using the Applied Biosystems high‐capacity RNA‐to‐cDNA kit (Life Technologies, UK), as per manufacturer's instructions. Real‐time quantitative polymerase chain reaction (PCR) was performed using Taqman Fast Universal PCR Mastermix (life technologies, UK). RNA expression levels of *Eotaxin‐2* (Mm00444701_m1, Applied biosytems) were normalised to GAPDH (Mm99999915_g1). The 2^−ΔΔCT^ relative quantitation method was used to determine the relative expression level of Eotaxin‐2 compared to control group.

### Statistics

All statistical analysis was performed with Prism version 5 software (GraphPad Software). Mann‐Whitney statistical test was used for comparison with wild‐type controls. Data presented as box and whisker plots, with the box showing the median and the 25th and 75th percentiles. Whiskers of the graph show the largest and the smallest values. *p values < 0.05, **p<0.01, ***p<0.001.

## Conflict of interest

The authors declare no financial or commercial conflict of interest.

**Abbreviation**
CD200R1: CD200 receptor

## Supporting information

Supplementary figure 1
Supplementary figure 2

## References

[1] Barclay, A. N. , Wright, G. J. , Brooke, G. and Brown, M. H. , CD200 and membrane protein interactions in the control of myeloid cells. Trends Immunol. 2002 23: 285–290. 12072366 10.1016/s1471-4906(02)02223-8

[2] Hoek, R. M. , Ruuls, S. R. , Murphy, C. A. , Wright, G. J. , Goddard, R. , Zurawski, S. M. , Blom, B. et al., Down‐regulation of the macrophage lineage through interaction with OX2 (CD200). Science 2000 290: 1768–1771. 11099416 10.1126/science.290.5497.1768

[3] Gorczynski, R. M. , Chen, Z. , Lee, L. , Yu, K. and Hu, J. , Anti‐CD200R ameliorates collagen‐induced arthritis in mice. Clin. Immunol. 2002 104: 256–264. 12217336 10.1006/clim.2002.5232

[4] Copland, D. A. , Calder, C. J. , Raveney, B. J. E. , Nicholson, L. B. , Phillips, J. , Cherwinski, H. , Jenmalm, M. et al., Monoclonal antibody‐mediated CD200 receptor signaling suppresses macrophage activation and tissue damage in experimental autoimmune uveoretinitis. Am. J. Pathol. 2007 171: 580–588. 17600119 10.2353/ajpath.2007.070272 PMC1934542

[5] Gorczynski, R. M. , Hu, J. , Chen, Z. , Kai, Y. and Lei, J. , A CD200FC immunoadhesin prolongs rat islet xenograft survival in mice. Transplantation 2002 73: 1948–1953. 12131694 10.1097/00007890-200206270-00018

[6] Nathan, C. and Muller, W. A. , Putting the brakes on innate immunity: a regulatory role for CD200? Nat. Immunol. 2001 2: 17–19. 11135572 10.1038/83124

[7] Hussell, T. and Bell, T. J. , Alveolar macrophages: plasticity in a tissue‐specific context. Nat. Rev. Immunol. 2014 14: 81–93. 24445666 10.1038/nri3600

[8] Snelgrove, R. J. , Goulding, J. , Didierlaurent, A. M. , Lyonga, D. , Vekaria, S. , Edwards, L. , Gwyer, E. , et al , A critical function for CD200 in lung immune homeostasis and the severity of influenza infection. Science 2008 9: 1074–1083. 10.1038/ni.1637 18660812

[9] Goulding, J. , Godlee, A. , Vekaria, S. , Hilty, M. , Snelgrove, R. and Hussell, T. , Lowering the threshold of lung innate immune cell activation alters susceptibility to secondary bacterial superinfection. J. Infect. Dis. 2011 204: 1086–1094. 21881124 10.1093/infdis/jir467 PMC3164429

[10] Mukhopadhyay, S. , Plüddemann, A. , Hoe, J. C. , Williams, K. J. , Varin, A. , Makepeace, K. , Aknin, M.‐L. et al., Immune inhibitory ligand CD200 induction by TLRs and NLRs limits macrophage activation to protect the host from meningococcal septicemia. Cell Host Microbe 2010 8: 236–247. 20833375 10.1016/j.chom.2010.08.005

[11] Soberman, R. J. , MacKay, C. R. , Vaine, C. A. , Ryan, G. B. , Cerny, A. M. , Thompson, M. R. , Nikolic, B. et al., CD200R1 supports HSV‐1 viral replication and licenses pro‐inflammatory signaling functions of TLR2. Ansari AA, ed. PLoS ONE. 2012 7:e47740. 23082204 10.1371/journal.pone.0047740 PMC3474780

[12] Stack, G. , Jones, E. , Marsden, M. , Stacey, M. A. , Snelgrove, R. J. , Lacaze, P. , Jacques, L. C. et al., CD200 receptor restriction of myeloid cell responses antagonizes antiviral immunity and facilitates cytomegalovirus persistence within mucosal tissue. Hill AB, ed. PLoS Pathog. 2015 11:e1004641. 25654642 10.1371/journal.ppat.1004641 PMC4412112

[13] Rygiel, T. P. , Rijkers, E. S. K. , de Ruiter, T. , Stolte, E. H. , van der Valk, M. , Rimmelzwaan, G. F. , Boon, L. et al., Lack of CD200 enhances pathological T cell responses during influenza infection. J. Immunol. 2009 183: 1990–1996. 19587022 10.4049/jimmunol.0900252

[14] Jarvis, J. N. and Harrison, T. S. , HIV‐associated cryptococcal meningitis. AIDS. 2007 21: 2119–2129. 18090038 10.1097/QAD.0b013e3282a4a64d

[15] Rohatgi, S. , Gohil, S. , Kuniholm, M. H. , Schultz, H. , Dufaud, C. , Armour, K. L. , Badri, S. et al., Fc gamma receptor 3A polymorphism and risk for HIV‐associated cryptococcal disease. MBio. 2013 4:e00573–13. 10.1128/mBio.00573-13 PMC3760251 23982074

[16] Singh, N. and Perfect, J. R. , Immune reconstitution syndrome associated with opportunistic mycoses. Lancet Infect Dis. 2007 7: 395–401. 17521592 10.1016/S1473-3099(07)70085-3

[17] Pappas, P. G. , Perfect, J. R. , Cloud, G. A. , Larsen, R. A. , Pankey, G. A. , Lancaster, D. J. , Henderson, H. et al., Cryptococcosis in human immunodeficiency virus‐negative patients in the era of effective azole therapy. Clin. Infect. Dis. 2001 33: 690–699. 11477526 10.1086/322597

[18] Park, B. J. , Wannemuehler, K. A. , Marston, B. J. , Govender, N. , Pappas, P. G. and Chiller, T. M. , Estimation of the current global burden of cryptococcal meningitis among persons living with HIV/AIDS. AIDS. 2009 23: 525–530. 19182676 10.1097/QAD.0b013e328322ffac

[19] Pfeffer, P. E. , Sen, A. , Das, S. , Sheaff, M. , Sivaramakrishnan, A. , Simcock, D. E. and Turner, B. , Eosinophilia, meningitis and pulmonary nodules in a young woman. Thorax. 2010 65: 1066–1085. 20889522 10.1136/thx.2010.140350

[20] Huffnagle, G. B. , Boyd, M. B. , Street, N. E. and Lipscomb, M. F. , IL‐5 is required for eosinophil recruitment, crystal deposition, and mononuclear cell recruitment during a pulmonary Cryptococcus neoformans infection in genetically susceptible mice (C57BL/6). J. Immunol. 1998 160: 2393–2400. 9498782

[21] Wright, G. J. , Cherwinski, H. , Foster‐Cuevas, M. , Brooke, G. , Puklavec, M. J. , Bigler, M. , Song, Y. et al., Characterization of the CD200 receptor family in mice and humans and their interactions with CD200. J. Immunol. 2003 171: 3034–3046. 12960329 10.4049/jimmunol.171.6.3034

[22] Hatherley, D. , Cherwinski, H. M. , Moshref, M. and Barclay, A. N. , Recombinant CD200 protein does not bind activating proteins closely related to CD200 receptor. J. Immunol. 2005 175: 2469–2474. 16081818 10.4049/jimmunol.175.4.2469

[23] Piehler, D. , Grahnert, A. , Eschke, M. , Richter, T. , Köhler, G. , Stenzel, W. and Alber, G. , T1/ST2 promotes T helper 2 cell activation and polyfunctionality in bronchopulmonary mycosis. Mucosal Immunol. 2013 6: 405–414. 22990621 10.1038/mi.2012.84

[24] Feldmesser, M. , Casadevall, A. , Kress, Y. , Spira, G. and Orlofsky, A. , Eosinophil‐Cryptococcus neoformans interactions in vivo and in vitro. Infect. Immun. 1997 65: 1899–1907. 9125578 10.1128/iai.65.5.1899-1907.1997 PMC175238

[25] Qiu, Y. , Dayrit, J. K. , Davis, M. J. , Carolan, J. F. , Osterholzer, J. J. , Curtis, J. L. and Olszewski, M. A. , Scavenger receptor A modulates the immune response to pulmonary Cryptococcus neoformans infection. J. Immunol. 2013 191: 238–248. 23733871 10.4049/jimmunol.1203435 PMC4007509

[26] Piehler, D. , Stenzel, W. , Grahnert, A. , Held, J. , Richter, L. , Köhler, G. , Richter, T. et al., Eosinophils contribute to IL‐4 production and shape the T‐helper cytokine profile and inflammatory response in pulmonary cryptococcosis. Am. J. Pathol. 2011 179: 733–744. 21699881 10.1016/j.ajpath.2011.04.025 PMC3157286

[27] Jarvis, J. N. , Casazza, J. P. , Stone, H. H. , Meintjes, G. , Lawn, S. D. , Levitz, S. M. , Harrison, T. S. et al., The phenotype of the Cryptococcus‐specific CD4+ memory T‐cell response is associated with disease severity and outcome in HIV‐associated cryptococcal meningitis. J. Infect. Dis. 2013 207: 1817–1828. 23493728 10.1093/infdis/jit099 PMC3654748

[28] Siddiqui, A. A. , Brouwer, A. E. , Wuthiekanun, V. , Jaffar, S. , Shattock, R. , Irving, D. , Sheldon, J. et al., IFN‐gamma at the site of infection determines rate of clearance of infection in cryptococcal meningitis. J. Immunol. 2005 174: 1746–1750. 15661940 10.4049/jimmunol.174.3.1746

[29] Jarvis, J. N. , Meintjes, G. , Bicanic, T. , Buffa, V. , Hogan, L. , Mo, S. , Tomlinson, G. et al., Cerebrospinal fluid cytokine profiles predict risk of early mortality and immune reconstitution inflammatory syndrome in HIV‐associated cryptococcal meningitis. Alspaugh JA, ed. PLoS Pathog. 2015 11:e1004754. 25853653 10.1371/journal.ppat.1004754 PMC4390200

[30] Wormley, F. L. , Perfect, J. R. , Steele, C. and Cox, G. M. , Protection against cryptococcosis by using a murine gamma interferon‐producing Cryptococcus neoformans strain. Infect. Immun. 2007 75: 1453–1462. 17210668 10.1128/IAI.00274-06 PMC1828544

[31] Uicker, W. C. , Doyle, H. A. , McCracken, J. P. , Langlois, M. and Buchanan, K. L. , Cytokine and chemokine expression in the central nervous system associated with protective cell‐mediated immunity against Cryptococcus neoformans. Med. Mycol. 2005 43: 27–38. 15712606 10.1080/13693780410001731510

[32] Zhou, Q. , Gault, R. A. , Kozel, T. R. and Murphy, W. J. , Protection from direct cerebral cryptococcus infection by interferon‐gamma‐dependent activation of microglial cells. J. Immunol. 2007 178: 5753–5761. 17442959 10.4049/jimmunol.178.9.5753

[33] Chang, C. C. , Omarjee, S. , Lim, A. , Spelman, T. , Gosnell, B. I. , Carr, W. H. , Elliott, J. H. et al., Chemokine levels and chemokine receptor expression in the blood and the cerebrospinal fluid of HIV‐infected patients with cryptococcal meningitis and cryptococcosis‐associated immune reconstitution inflammatory syndrome. J. Infect. Dis. 2013 208: 1604–1612. 23908492 10.1093/infdis/jit388 PMC3805241

[34] Boulware, D. R. , Bonham, S. C. , Meya, D. B. , Wiesner, D. L. , Park, G. S. , Kambugu, A. , Janoff, E. N. et al., Paucity of initial cerebrospinal fluid inflammation in cryptococcal meningitis is associated with subsequent immune reconstitution inflammatory syndrome. J. Infect. Dis. 2010 202: 962–970. 20677939 10.1086/655785 PMC2924457

[35] Longley, N. , Harrison, T. S. and Jarvis, J. N. , Cryptococcal immune reconstitution inflammatory syndrome. Curr. Opin. Infect. Dis. 2013 26: 26–34. 23242412 10.1097/QCO.0b013e32835c21d1

[36] Meya, D. B. , Manabe, Y. C. , Boulware, D. R. and Janoff, E. N. , The immunopathogenesis of cryptococcal immune reconstitution inflammatory syndrome: understanding a conundrum. Curr. Opin. Infect. Dis. 2016 29: 10–22. 26658650 10.1097/QCO.0000000000000224 PMC4689618

[37] Humphreys, I. R. , Walzl, G. , Edwards, L. , Rae, A. , Hill, S. and Hussell, T. , A critical role for OX40 in T cell‐mediated immunopathology during lung viral infection. J. Exp. Med. 2003 198: 1237–1242. 14568982 10.1084/jem.20030351 PMC2194232

[38] Murdock, B. J. , Teitz‐Tennenbaum, S. , Chen, G.‐H. , Dils, A. J. , Malachowski, A. N. , Curtis, J. L. , Olszewski, M. A. et al., Early or late IL‐10 blockade enhances Th1 and Th17 effector responses and promotes fungal clearance in mice with cryptococcal lung infection. J. Immunol. 2014 193: 4107–4116. 25225664 10.4049/jimmunol.1400650 PMC4193595

[39] Leopold Wager, C. M. , Hole, C. R. , Wozniak, K. L. , Olszewski, M. A. , Mueller, M. and Wormley, F. L. , STAT1 signaling within macrophages is required for antifungal activity against Cryptococcus neoformans. Deepe GS Jr., ed. Infect. Immun. 2015 83: 4513–4527. 26351277 10.1128/IAI.00935-15 PMC4645398

[40] Qiu, Y. , Zeltzer, S. , Zhang, Y. , Wang, F. , Chen, G.‐H. , Dayrit, J. , Murdock, B. J. et al., Early induction of CCL7 downstream of TLR9 signaling promotes the development of robust immunity to cryptococcal infection. J. Immunol. 2012 188: 3940–3948. 22422883 10.4049/jimmunol.1103053 PMC3324623

[41] Hardison, S. E. , Ravi, S. , Wozniak, K. L. , Young, M. L. , Olszewski, M. A. and Wormley, F. L. , Pulmonary infection with an interferon‐gamma‐producing Cryptococcus neoformans strain results in classical macrophage activation and protection. Am. J. Pathol. 2010 176: 774–785. 20056835 10.2353/ajpath.2010.090634 PMC2808084

[42] Monticelli, L. A. , Sonnenberg, G. F. , Abt, M. C. , Alenghat, T. , Ziegler, C. G. K. , Doering, T. A. , Angelosanto, J. M. et al., Innate lymphoid cells promote lung‐tissue homeostasis after infection with influenza virus. Nat. Immunol. 2011 12: 1045–1054. 21946417 10.1031/ni.2131 PMC3320042

[43] Gold, M. J. , Antignano, F. , Halim, T. Y. F. , Hirota, J. A. , Blanchet, M.‐R. , Zaph, C. , Takei, F. et al., Group 2 innate lymphoid cells facilitate sensitization to local, but not systemic, TH2‐inducing allergen exposures. J. Allergy Clin. Immunol. 2014 133: 1142–1148. 24679471 10.1016/j.jaci.2014.02.033

[44] Christianson, C. A. , Goplen, N. P. , Zafar, I. , Irvin, C. , Good, J. T. , Rollins, D. R. , Gorentla, B. et al., Persistence of asthma requires multiple feedback circuits involving type 2 innate lymphoid cells and IL‐33. J. Allergy Clin. Immunol. 2015 136: 59–68.e14. 25617223 10.1016/j.jaci.2014.11.037 PMC4494983

