# Supplementary material for: CD200R1 regulates eosinophilia during pulmonary fungal infection in mice
Source: Eur J Immunol. 2019 Aug 7;49(9):1380–90. doi: 10.1002/eji.201847861 (PMC6773205; doi:10.1002/eji.201847861)

European Journal of Immunology

Supporting Information
for
DOI 10.1002/eji.201847861

Samira Salek-Ardakani, Thomas Bell, Christopher P Jagger, Robert J Snelgrove and Tracy Hussell

CD200R1 regulates eosinophilia during pulmonary fungal infection in mice

© 2019 The Authors. *European Journal of Immunology* published by WILEY-VCH Verlag GmbH & Co. KGaA, Weinheim.

Supplementary Figure 1

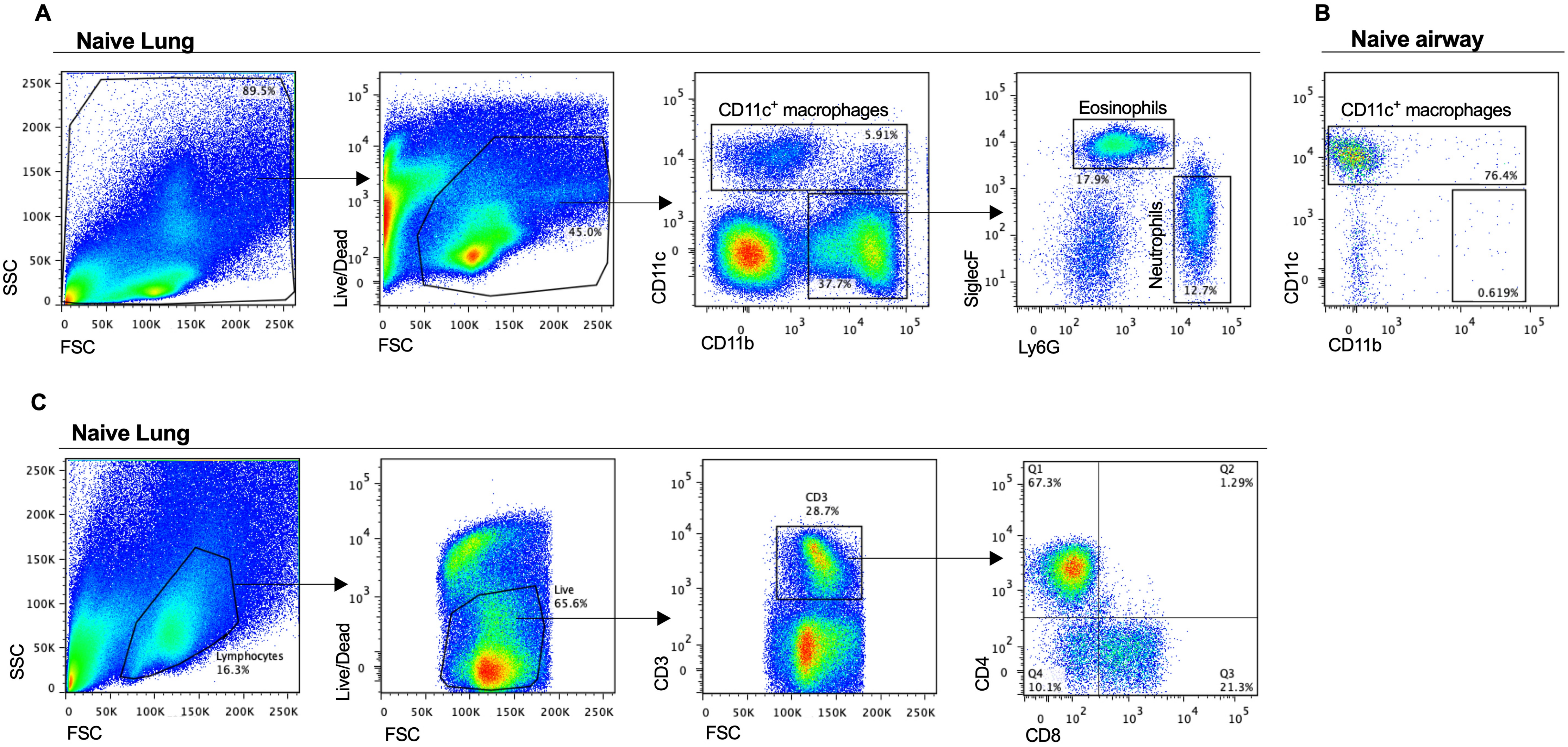

Supplementary Figure 2

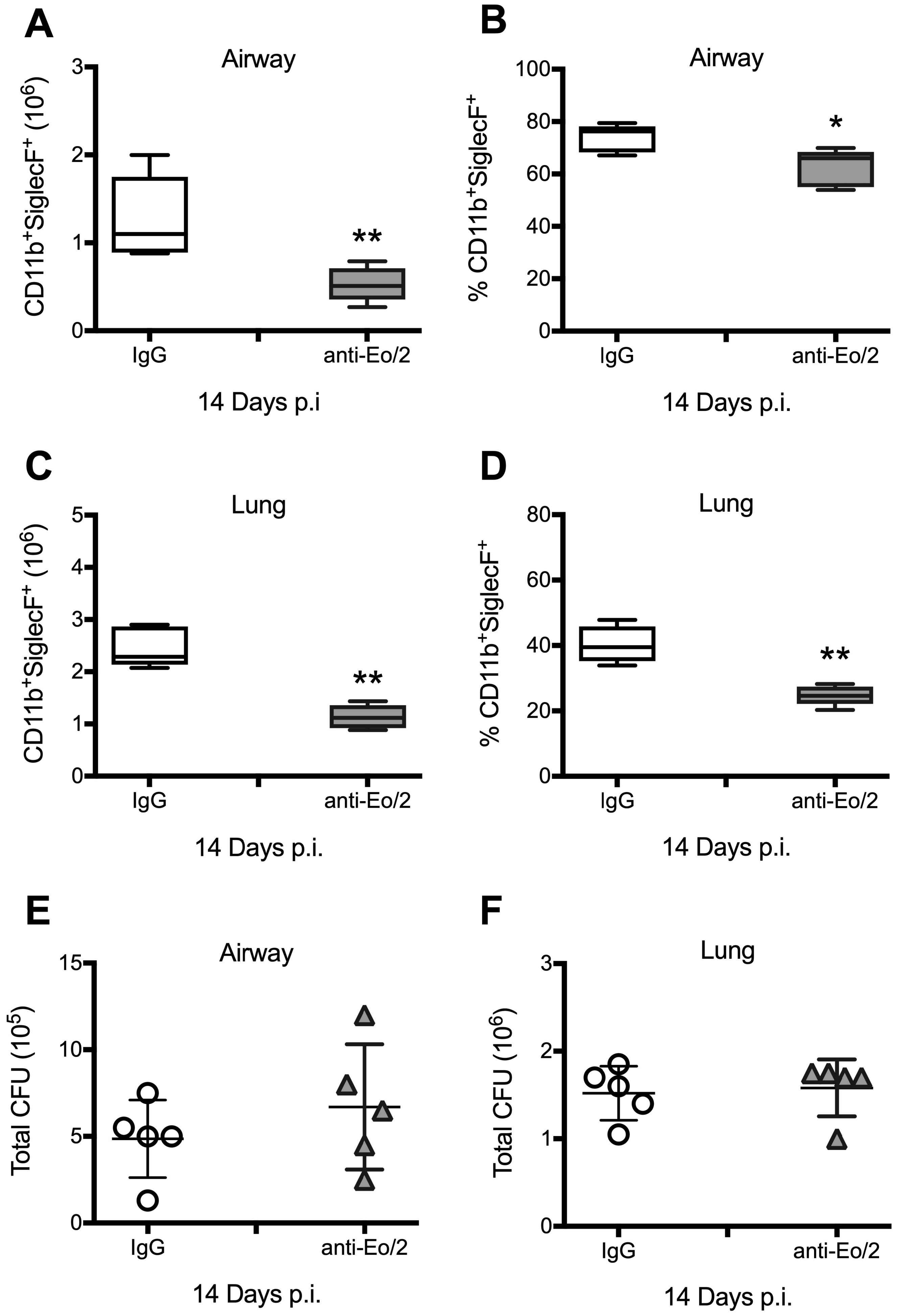

Supplement: Supplementary file 1 — Supplementary figure 1 Supplementary figure 2 [file EJI-49-1380-s001.pdf]
